# The Carolina hysterectomy cohort (CHC): a novel case series of reproductive-aged hysterectomy patients across 10 hospitals in the US south

**DOI:** 10.1186/s12905-023-02837-8

**Published:** 2023-12-19

**Authors:** Zakiya M. Haji-Noor, Joacy G. Mathias, Theo Gabriel Beltran, Lauren G. Anderson, Mollie E. Wood, Annie Green Howard, Sharon Peacock Hinton, Kemi M. Doll, Whitney R. Robinson

**Affiliations:** 1 Department of Epidemiology, University of North Carolina at Chapel Hill, Chapel Hill, NC USA; 2 Division of Women’s Community and Population Health and Department of Obstetrics Gynecology, Duke University School of Medicine, Durham, North, Carolina USA; 3 Department of Biostatistics, University of North Carolina at Chapel Hill, Chapel Hill, NC USA; 4 Carolina Population Center, University of North Carolina at Chapel Hill, Chapel Hill, NC USA; 5 Department of Obstetrics & Gynecology, University of Washington School of Medicine, Seattle, WA USA; 6 Department of Health Services, University of Washington School of Public Health, Seattle, WA USA

**Keywords:** Reproductive health, Hysterectomy, Electronic medical record, Health disparity, Epidemiology, Case series, Cohort profile

## Abstract

**Background:**

Hysterectomy is a common surgery among reproductive-aged U.S. patients, with rates highest among Black patients in the South. There is limited insight on causes of these racial differences. In the U.S., electronic medical records (EMR) data can offer richer detail on factors driving surgical decision-making among reproductive-aged populations than insurance claims-based data. Our objective in this cohort profile paper is to describe the Carolina Hysterectomy Cohort (CHC), a large EMR-based case-series of premenopausal hysterectomy patients in the U.S. South, supplemented with census and surgeon licensing data. To demonstrate one strength of the data, we evaluate whether patient and surgeon characteristics differ by insurance payor type.

**Methods:**

We used structured and abstracted EMR data to identify and characterize patients aged 18–44 years who received hysterectomies for non-cancerous conditions between 10/02/2014–12/31/2017 in a large health care system comprised of 10 hospitals in North Carolina. We used Chi-squared and Kruskal Wallis tests to compare whether patients’ socio-demographic and relevant clinical characteristics, and surgeon characteristics differed by patient insurance payor (public, private, uninsured).

**Results:**

Of 1857 patients (including 55% non-Hispanic White, 30% non-Hispanic Black, 9% Hispanic), 75% were privately-insured, 17% were publicly-insured, and 7% were uninsured. Menorrhagia was more prevalent among the publicly-insured (74% vs 68% overall). Fibroids were more prevalent among the privately-insured (62%) and the uninsured (68%). Most privately insured patients were treated at non-academic hospitals (65%) whereas most publicly insured and uninsured patients were treated at academic centers (66 and 86%, respectively). Publicly insured and uninsured patients had higher median bleeding (public: 7.0, uninsured: 9.0, private: 5.0) and pain (public: 6.0, uninsured: 6.0, private: 3.0) symptom scores than the privately insured. There were no statistical differences in surgeon characteristics by payor groups.

**Conclusion:**

This novel study design, a large EMR-based case series of hysterectomies linked to physician licensing data and manually abstracted data from unstructured clinical notes, enabled identification and characterization of a diverse reproductive-aged patient population more comprehensively than claims data would allow. In subsequent phases of this research, the CHC will leverage these rich clinical data to investigate multilevel drivers of hysterectomy in an ethnoracially, economically, and clinically diverse series of hysterectomy patients.

**Supplementary Information:**

The online version contains supplementary material available at 10.1186/s12905-023-02837-8.

## Introduction

Every year in the US, approximately 600,000 patients undergo a hysterectomy—a surgery during which the uterus is removed [1, 2]. An estimated 20 million patients in the US have had a hysterectomy [1, 3, 4]. Hysterectomies are often performed for benign gynecologic conditions such as fibroids, endometriosis, pelvic pain, and uterine prolapse [5]. After cesarean sections, hysterectomies are the second most common surgery performed on US women under age 65 with one-third receiving hysterectomy before age 60 [6].While the surgery can be an effective treatment for debilitating gynecologic conditions and lowers endometrial cancer risk, there are severe, irreversible consequences such as infertility, surgical complications, and increased stroke and mortality risks to consider especially when operating for benign gynecological conditions [7–10].

Most studies of gynecologic treatments like hysterectomy are unable to account for the treatment trade-offs and complex decision-making described above. A major reason is that these studies fail to capture multilevel influences on hysterectomy decision-making in diverse, population-based samples [11, 12]. Multilevel influences include patient factors (e.g., symptom severity, sociodemographic factors, other clinical factors), clinician factors (e.g., sociodemographics, clinical experience), and health care setting factors (e.g., type of practice, case mix, insurance mix). For instance, population-based studies using insurance claims or national databases, such as the National Inpatient Sample and National Hospital Discharge System, cannot measure key patient clinical factors, such as the symptom severity of benign gynecologic diseases, or important potentially important provider and health system factors [13]. Finally, insurance claims-based studies are often limited to single payor types, leaving the uninsured rarely studied [14–18]. In contrast to all the limitations mentioned above, EMR-based studies have rich data on patient, provider, and health care factors. However, like insurance-claims-based studies, EMR-based studies from single hospitals or small health systems fail to capture the breadth of people affected by gynecologic conditions [19–21].

In this paper, first, we describe a novel approach to overcome these barriers. We leverage multiple data sources to supplement EMR data from a large (*n* > 1800), population-based series of hysterectomy patients treated in a large health system in a southeastern state. We supplemented structured EMR data with manual EMR abstraction to collect in-depth patient clinical data unavailable in diagnostic codes. We also linked these data to surgeon-level licensing data and patient residential census data. Second, to demonstrate one strength of the data, we evaluated whether patient and surgeon characteristics differed between the uninsured (who are not represented in insurance claims-based analyses) and the rest of the population..

## Materials and methods

### Study design

This study consists of a case series of patients who underwent hysterectomy between October 2nd, 2014, and December 31st, 2017, in one of 10 hospitals that were part of a large health care system in one southeastern state. Analysis of this growing system, comprising academic centers and large and small community hospitals, is an enormous strength, allowing us to examine the heterogeneity of care. This healthcare system has a presence in 100 counties in the state, extending into neighboring states, including metro areas as well as suburban and rural areas. One of the hospitals is an academic medical center that serves a high proportion of uninsured patients in addition to the privately and publicly insured population.

**Fig. 1:**
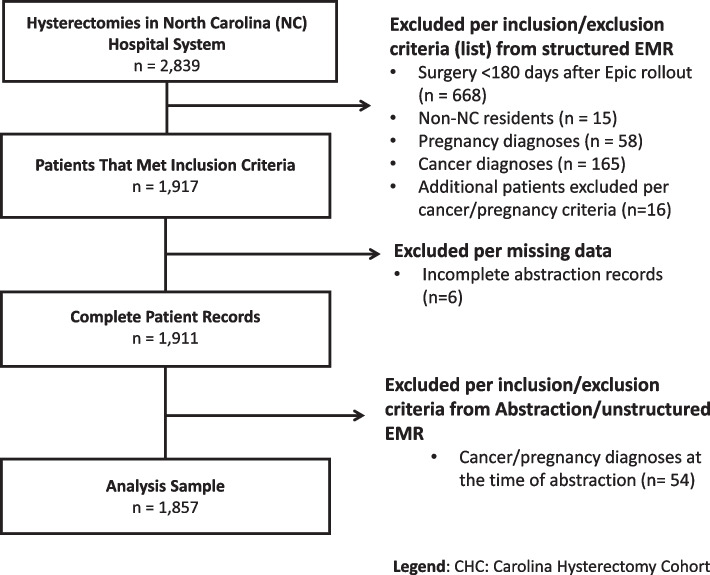
Flow chart of inclusion/exclusion criteria of CHC, ages 18 to 44 years undergoing surgery between 10/02/2014–12/31/2017

Individuals were included if they underwent hysterectomy for a benign gynecologic condition between ages 18 and 44 years (see Fig. 1 for exclusion criteria). To ensure a sufficient lookback period (6 months) of patients’ gynecological history, patients were excluded if their surgery occurred less than 6 months after the rollout of the EMR system (*n* = 668). Patients were also excluded if they were pregnant at the time of surgery (*n* = 58), had prior or active breast, ovarian, uterine, or cervical cancer diagnoses, or diagnoses for other cancers whose treatment plans may involve hysterectomy (bladder, anal, colorectal) (*n* = 165).

### Data sources

**Fig. 2:**
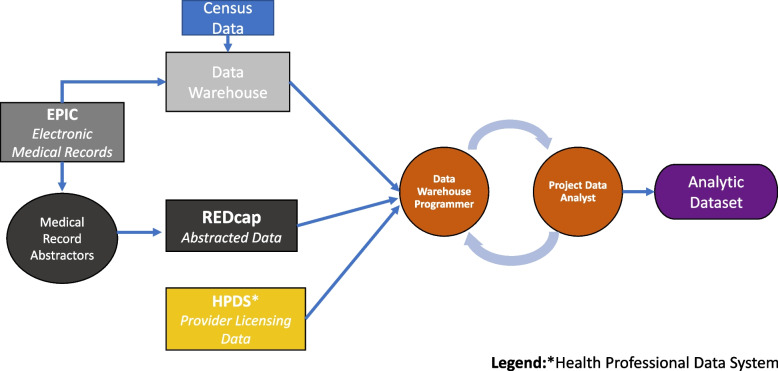
Data flow from various sources used to derive the Carolina Hysterectomy Cohort analytical dataset

The parent study linked data from the healthcare system, the U.S. census, and the state physician licensing board to develop a multifaceted approach for understanding determinants of treatment with hysterectomy (see Fig. 2).

#### Electronic medical record data

Structured EMR data – Data Warehouse: The health care system licenses a data warehouse to facilitate access to EMR data for research. Honest broker analysts from the data warehouse identified the study population by searching the EMR for hysterectomy procedure codes and relevant diagnostic codes provided by the research team. The data warehouse also provisioned to our team structured data on those patients, including patient demographics, diagnoses and procedures associated with the surgery, and other pre-specified treatments, prescriptions, and health care encounters.

EMR free text – Medical Record Abstraction: Additionally, we leveraged the unstructured EMR data to abstract detailed clinical information. After a thorough pilot study to refine an EMR abstraction process, the study team created a comprehensive abstraction protocol and data collection tool with Research Electronic Data Capture (REDCap) to input patient data. The EMR abstraction protocol, REDCap data collection tool, and accompanying data codebook are freely available for review [22–24]. Using the tools mentioned above, a team of professional medical record abstractors reviewed surgeon-reported patient progress and operative notes, and imaging and pathology reports. The information collected from the surgeon-reported notes included the presence or absence of patient symptom and diagnoses, previous treatments, imaging and pathology report findings, primary assessments of reason for surgery, and notes from the surgical operative report. The abstraction process and validation are described in more detail by Doll, et al. [25].

#### Census data

Finally, the Data Warehouse linked geocoded addresses of hysterectomy patients at the time of surgery, to U.S. census tract data. Census data from the U.S. Decennial Census and ongoing 2014 American Community Survey provided data about patients’ residential contexts.

### Payor grouping

We grouped patients by payor according to the following categories: public, private, and uninsured. Public includes patients covered by Medicare (*N* = 67), Medicaid (*N* = 226), or receiving care at a prison facility. Private includes those with private insurance (*N* = 1340) or Tricare (*N* = 53), coverage provided to military service members and their families. Finally, the uninsured category includes those patients whose records indicated “self-pay,” either partially (*n* < 10) or wholly (*n* = 141).

### Medical conditions

Because we performed medical record abstraction on all surgeries, we were able to classify surgeries by associated gynecologic conditions in two ways: (1) all gynecologic diagnoses listed at time of surgery and (2) the primary indication indicated in the surgeon’s pre-operative note. The former represents formal diagnoses associated with the surgery that have been identified using ICD-9 and ICD-10 codes and is commonly used in claims-based research. The latter, the primary reason for the surgery that the surgeon recorded in the text of the pre-operative note, is often not available in insurance claims. While both categorization systems allow for multiple conditions to be listed, the list of gynecologic diagnoses can be quite long, while the list of primary indications from the pre-operative note was usually limited to 1 or 2 conditions.

### Patient symptom severity scores

We scored each patient on severity of their gynecologic symptoms to create a composite severity index on three domains: bulk, vaginal bleeding, and pelvic pain [25]. Example candidate input factors for Bulk score (score range 0 to 39) were bloating (1 point), pelvic pressure (1 point), bulk diagnosis at surgery, or preoperatively (2–3 points) and uterine size (4 points). The candidate input factor examples for bleeding score (score range 0 to 26) were vaginal bleeding (1 point), period for more than 7 days (2 points), anemia (4 points) and a history of blood transfusion (5 points). Example candidate input factors for pain score (score range 0 to 14) were pelvic pain (1 point), pain diagnosis code in the year before surgery (3 points), and opioid usage (4 points).

### Linking surgeon data

**Fig. 3:**
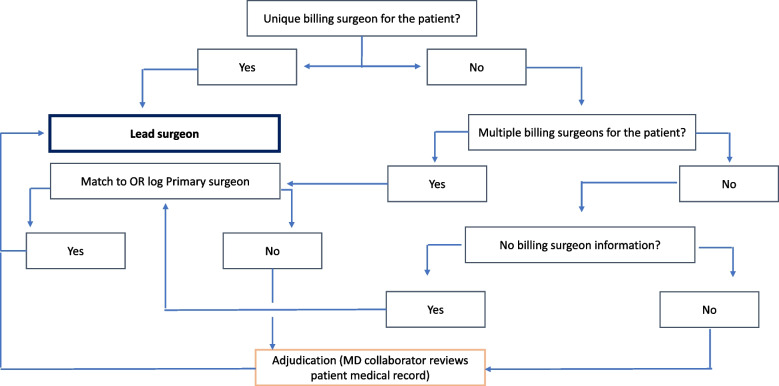
Algorithm showing how the lead surgeon was identified for each patient who underwent hysterectomy between 10/02/2014–12/31/2017

We merged surgeon demographic and occupational data with the CHC patient dataset to understand practice associations with hysterectomy decision-making. We obtained surgeon data from a state specific Health Professions Dataset (HPDS). The HPDS maintains and disseminates the licensed medical professionals’ demographics (e.g., gender, race, ethnicity), education (e.g., graduation year, where trained,) and practice-level data (practice setting, total hours worked in an average week and percent time in direct patient care). The HPDS has produced and maintained continuous data files since 1979. We identified and linked the primary surgeon’s information to the patient data. See Fig. 3 for the primary surgeon identification algorithm. Briefly, the patient’s billing surgeon was identified as the lead surgeon. If the patient had multiple billing surgeons, then the lead surgeon was the surgeon who was listed as the primary surgeon in the OR log. An MD collaborator reviewed the algorithm results and adjudicated and confirmed the lead surgeon from reviewing patient records. Using practice name and address, we grouped all surgeons by the practices in which they work. The linked physician licensing data will be used to create surgeon- and practice-level variables, including surgeon volume but also novel measures such as distinctive practice-level treatment patterns. These variables will be utilized in future studies.

### Analysis

We performed descriptive analyses in which we present counts and frequencies stratified by insurance payor. We used chi-squared/Fisher’s exact and Kruskal-Wallis to test whether the patient and surgeon characteristics differed by insurance payor. All analyses were performed using the Statistical Analysis System (SAS) statistical software package, version 9.4. SAS Institute Inc., Cary, NC, USA.

## Results

### Description of overall cohort: Carolina hysterectomy cohort

We identified 1857 patients through the EMR who underwent hysterectomy for benign gynecologic conditions and fit inclusion criteria described earlier. As shown in Table 1, most were non-Hispanic White (55.5%); about a third were non-Hispanic Black (30.4%); and the remaining were Hispanic (8.5%), non-Hispanic Asian (1.2%), American Indian/Alaskan Native (0.9%), or identified as other or unknown/refused (3.5%). In our case series, 57.2% were married and 73.8% lived in urban areas. They had a variety of insurance coverage types: public (17.3%), private (75.0%), and uninsured (7.7%) (Table 1). While the majority of patients were treated at a non-academic hospital (57.8%), almost half (42.2%) were treated at an academic medical center.

**Table 1:** Comparison of characteristics of EMR-based CHC versus stratified sample based on payor between 10/02/2014–12/31/2017

Variable	Categories	EMR based total Sample	Public^a^	Private^a^	Uninsured^a^		*p*-value
		N (%) / Median (IQR) *N* = 1857 (100%)	N (column percent)	N (column percent)	N (column percent)		
			*N* = 321 (100%)	*N* = 1393 (100%)	*N* = 143 (100%)		
**DEMOGRAPHIC CHARACTERISTICS**							
Race/ethnicity^e^	Non-Hispanic White	1030 (55.5)	174 (54.2)	814 (58.4)	42 (29.4)		<.0001
	Non-Hispanic African American/Black	565 (30.4)	111 (34.6)	426 (30.6)	28 (19.6)		
	Hispanic	158 (8.5)	18 (5.6)	75 (5.4)	65 (45.5)		
	Non-Hispanic Asian	22 (1.2)	< 10	19 (1.4)	< 10		
	Non-Hispanic American Indian/Alaska Native	17 (0.9)	< 10	< 10	< 10		
	Other	31 (1.7)	< 10	25 (1.8)	< 10		
	Unknown/Refused	34 (1.8)	< 10	25 (1.8)	< 10		
Marital status^e^	Married	1063 (57.2)	93 (29.0)	903 (64.8)	67 (46.9)		<.0001
	Single	509 (27.4)	144 (44.9)	306 (22.0)	59 (41.3)		
	Divorced	128 (6.9)	37 (11.5)	85 (6.1)	< 10		
	Legally Separated	52 (2.8)	15 (4.7)	33 (2.4)	< 10		
	Widowed	< 10	< 10	< 10	< 10		
	Domestic Partner	< 10	< 10	< 10	< 10		
	Unknown	98 (5.3)	29 (9.0)	63 (4.5)	< 10		
Age at Hysterectomy (y)^f^	*Median (IQR)*	40.1 (36.6, 42.8)	38.8 (33.4, 42.2)	40.3 (36.9, 42.9)	40.1 (36.9, 43.2)		<.0001
Rurality of patient’s home address^bg^	Urban	1371 (73.8)	204 (63.6)	1062 (76.2)	105 (73.4)		<.0001
	Rural	486 (26.2)	117 (36.5)	331 (23.8)	38 (26.6)		
**CLINICAL CONDITIONS**							
Diagnoses associated with surgery^cg^	Menorrhagia	1266 (68.2)	236 (73.5)	934 (67.0)	96 (67.1)		0.0776
	Fibroids	1104 (59.5)	150 (46.7)	857 (61.5)	97 (67.8)		<.0001
	Endometriosis	659 (35.5)	113 (35.2)	487 (35.0)	59 (41.3)		0.3229
	Anemia	359 (19.3)	61 (19.0)	254 (18.2)	44 (30.8)		0.0014
	Pain	298 (16.0)	76 (23.7)	200 (14.4)	22 (15.4)		0.0002
Primary indication for hysterectomy, according to surgeon pre-operative note^dg^	Menorrhagia	640 (34.5)	76 (23.7)	542 (38.9)	22 (15.4)		<.0001
	Fibroids	692 (37.3)	80 (24.9)	556 (39.9)	56 (39.2)		<.0001
	Abnormal uterine bleeding/ dysfunctional uterine bleeding/menometrorrhagia	472 (25.4)	134 (41.7)	286 (20.5)	52 (36.4)		<.0001
	Dysmenorrhea	326 (17.6)	70 (21.8)	231 (16.6)	25 (17.5)		0.0855
Charlson’s comorbidity index		0 (0, 0)	0 (0, 1)	0 (0, 0)	0 (0, 0)		<.0001
**SYMPTOM SEVERITY SCORES**							
Bulk^f^	*Median (IQR)*	1.0 (0.0,4.0)	1.0 (0.0, 2.0)	1.0 (0.0, 4.0)	1.0 (0.0, 4.0)		0.0013
Bleed^f^	*Median (IQR)*	5.0 (2.0,10.0)	7.0 (3.0, 12.0)	5.0 (2.0, 9.0)	9.0 (4.0, 19.0)		<.0001
Pain^f^	*Median (IQR)*	4.0 (1.0, 8.0)	6.0 (3.0, 11.0)	3.0 (0.0, 7.0)	6.0 (2.0, 11.0)		<.0001
**HOSPITAL**							
Hospital type^g^	Non-Academic Medical Center	1073 (57.8)	122 (38.0)	931 (66.8)	20 (14.0)		<.0001
	Teaching/Academic Medical Center	784 (42.2)	199 (62.0)	462 (33.2)	123 (86.0)		
**SURGEON CHARACTERISTICS (N= 115)**							
Sex^e^	Female		63 (54.8)	11 (37.9)	35 (45.5)	< 10	0.3271
	Male		52 (45.2)	18 (62.1)	42 (54.6)	< 10	
Race/Ethnicity^e^	Non-Hispanic White		82 (71.3)	21 (72.4)	53 (68.8)	< 10	0.9695
	Non-Hispanic Black		11 (9.6)	< 10	< 10	< 10	
	Asian/Pacific Islander		12 (10.4)	< 10	< 10	< 10	
	Hispanic		< 10	< 10	< 10	< 10	
Surgeon volume^e^	Annualized surgeon volume < 10		97 (84.3)	25 (86.2)	64 (83.1)	< 10	0.8586
	Annualized surgeon volume > =10		18 (15.7)	< 10	13 16.88	< 10	
Surgeon years in practice since June 30 of residency completion year	Median (IQR)		13.5 (3.5, 21.0)	10.0 (3.0, 21.0)	15.0 (6.0, 21.0)	10.0 (1.0, 17.0)	0.5049
Surgeon reported primary area of practice^e^	Gynecology/Gynecologic oncology		*28 (24.4)*	< 10	19 (24.7)	< 10	0.0003
	Obstetrics and Gynecology		*85 (73.9)*	21 (72.4)	58 (75.3)	< 10	
	Other		*< 10*	< 10	< 10	< 10	

^a^Public includes those who are covered by Medicaid (*N* = 226), Medicare (*N* = 67), dual eligibility, and state-covered incarcerated patients (*N* = 28); private includes those who are covered by private insurance (*N* = 1340), including those who have combination coverage, and Tricare (*N* = 53); uninsured (*N* = 143) includes those who have no other coverage; ^b^Patients were excluded if they were not residents of the state in which the hospital system is based either at the time of surgery or at the time of data pull; ^c^Diagnoses listed in patients’ structured EMR at time of surgery; they are neither exclusive (total > 1911) nor exhaustive; the ones included here are the most prevalent in this sample, present in at least 298 patients (16%); ^d^ Preoperative primary indication for surgery from unstructured EMR; The categories are not mutually exclusive. In addition, the categories presented are not exhaustive: instead, the ones included here are the most prevalent in this sample, present in at least 326 patients (17.6%); ^e^ Exact test; ^f^ Kruskal-Wallis test; ^g^ Chi-square test; *IQR* Interquartile range, *CHC* Carolina Hysterectomy Cohort

The most common diagnosis, identified using diagnostic codes from the structured EMR, associated with surgery from the data warehouse was menorrhagia (68.2%). However, a larger proportion (73.5%) of publicly insured patients had diagnoses of menorrhagia. The second most common diagnostic code among the overall sample was fibroids (59.5%), with larger proportions of private (61.5%) and uninsured (67.8%) patients having fibroid diagnoses than the overall sample.

We identified 25 unique practices and 115 primary surgeons who worked in these practices during the study period. More than half of the surgeons identified as female (54.8%) with a median age of 43 years (IQR: 34, 50 – data not shown). The median age of male surgeons was 49 years (IQR: 40, 56 – data not shown). Most surgeons were non-Hispanic White (71.3% were non-Hispanic White, 10.4% were Asian/ Pacific Islanders, 8.7 were non-Hispanic Black, 7.8% were Hispanic, and the rest identified as “Other.”) The median annualized surgeon volume was 4 (IQR: 2, 7.5 – data not shown). Over 75% of the surgeons had an annualized volume of 10 or less. Over 90% of the surgeons reported Gynecology, Gynecologic Oncology, or Obstetrics and Gynecology as their primary practice areas.

### Comparisons between uninsured patients and the rest of the hysterectomy sample

Patient sociodemographic characteristics differed by payor. Hispanic patients were disproportionately likely to be uninsured: while they only represented 8.5% of the total population, they represented almost half (45.5%) of the uninsured population. For White and Black patients, the likelihood of being privately insured was similar, although Black patients were slightly more likely to be publicly insured. Among the uninsured, 41.3% were single and among the publicly insured, 44.9%, whereas among the overall population, only 27.4% were single. Married patients, who comprised 57.2% of the whole population, were disproportionately likely to be privately insured, comprising 64.8% of that population.

The uninsured and the publicly insured patients had higher median bleeding (9.0 (4.0, 19.0) and 7.0 (3.0, 12.0), respectively) scores than the privately insured. The median pain scores (6.0 (3.0, 11.0 and 6.0 (3.0, 11.0, respectively) in the uninsured and publicly insured were similar although higher than the privately insured (3.00 (0, 7.0)). The Charlson’s comorbidity index (CCI) in these mostly premenopausal benign patients was highly skewed. Most patients had a score of 0, unlike studies of older hysterectomy patients or those receiving hysterectomy as treatment for endometrial cancer [26].

In the overall patient population, based on the abstracted pre-operative EMR notes, the most common surgeon-reported reason for surgery was fibroids, a main reason for 37.3% of patients. Menorrhagia was also a common indication, listed in notes as a main reason for surgery in 34.5% of patients. However, the patterns differed for uninsured patients. Fibroids were the most common indication (39.2%) for the uninsured, but the second most common was abnormal uterine bleeding (36.4%), with menorrhagia only noted as a main indication in 15.4% of uninsured patients. Surgeon characteristics such as sex, race/ethnicity, surgeon volume and time since residency, did not statistically differ by insurance payor groups.

## Discussion

Leveraging data from a healthcare delivery system allowed us to identify a diverse case series of patients using EMR data from a single-state health care system. Our study sample includes a reproductive-aged patient population that is diverse with respect to race/ethnicity, insurance status, and residential environments. Due to our focus on premenopausal hysterectomy, our study population is younger when compared with the state-wide claims based hysterectomy population, however, the demographic characteristics are similar [17]. Reproductive-aged Hispanic and Black patients were disproportionately likely to be uninsured. Comparing uninsured patients to other payor groups, we found differences in their marital status, primary reasons for surgery and symptom severity.

An analysis using claims data instead of EMR data would limit analyses to insured (single payor) or Medicaid patients. As a result, these claims-based analyses would disproportionately exclude Black and Hispanic patients from study, further exacerbating health inequities, some of which may be invisible in studies using EMR alone.

This study demonstrates a feasible method for capturing a reproductive-aged population that is more diverse than that captured from private insurance claims data and/or Medicaid data alone. A large issue that remains unaddressed with private insurance claims and Medicaid is the large proportion of adults in the USA who are uninsured—approximately 12.4% of adults (in 2016) [27]. Indeed, in our population of hysterectomy patients, 7.7% were uninsured. This group were disproportionately likely to be Hispanic, single, and live in a census tract with lower median household income than the overall population. Use of claims renders the uninsured absent from research. The ability to achieve more complete condition identification is an advantage of EMR data over claims data [18].

Another advantage of EMR-based research on reproductive-aged populations over claims-based research and National Inpatient and National Hospital Discharge datasets is the richness of unstructured patient EMR data. The primary indications abstracted from the unstructured EMR gave us more specificity about reasons for surgery than diagnostic codes associated with surgery. For example, we found that most patients had multiple diagnostic codes associated with their surgeries, making it difficult to distinguish important clinical indications for a hysterectomy. In contrast, abstracting from the preoperative notes helped focus on 1 or 2 important indications for the surgery. Additionally, the National Inpatient Sample is not representative of hysterectomy patient population as a great majority of hysterectomy now happens in outpatient settings [28].

The main operational challenges we encountered revolved around the need to harmonize structured and abstracted data from electronic medical records while also managing variations in EMR rollout dates. There were also some discrepancies in the structured EMR, which relied on ICD-9/ICD-10 codes, when compared to the unstructured EMR, which was abstracted from notes into REDCap. While this required additional time, labor/personnel, and costs for data cleaning and data management, this enabled us to capture a more detailed picture of how and when encounters happened as well as additional clinical detail when compared to structured EMR data alone. Additionally, we faced dynamic administrative circumstances as hospitals had variable EMR rollout dates. A primary exclusion criterion of our case series is a patient lookback period of 180 days, which resulted in the exclusion of 668 patients due to insufficient lookback period. During the time of patient selection, one site was acquired by an entity external to the health system under study, so this particular site only contributed patients until September 1st, 2018, varying from other sites.

Our study is limited to gynecologic surgery patients within one healthcare system in the U.S. South. Results may differ in larger or other kinds of health systems or for other health conditions. As with claims research, data are often reported by surgeons rather than from the patients themselves. As a consequence, some key variables, such as race, may differ from what patients would self-report themselves. Further research could supplement EMR-derived quantitative study designs with qualitative work or with direct patient surveys. We also were underpowered to examine hysterectomy among Asian-American, American Indian, and individuals of other racial or ethnic groups. In particular, Asian-Americans had very low numbers of hysterectomy in our sample, reflecting a relatively small proportion within the catchment area for this health system and their low rates of hysterectomy in the South [29]. An additional challenge with studying American Indian populations is that health services databases typically have low sensitivity for identifying them [30]. However, we believe that these descriptive analyses in these groups will serve as a foundation for future research for these groups, who are often, unfortunately, excluded from gynecologic health research. Much health services research that investigates influences on outcomes focuses on patient individual and residential environments and neglects multilevel health care factors.

## Conclusion

We demonstrated that it is possible to assemble a socioeconomically, racial/ethnically and clinically diverse patient cohort of premenopausal patients using EMR data and link that patient population to surgeon and health care setting data. By considering differences in a single state, we minimized confounding by regional variation in practice. Rather than relying solely on diagnostic codes at single clinical sites, we leveraged richer clinical data. Finally, we included uninsured patients and demonstrated that these patients differ sociodemographically and clinically from insured patients. Other studies that exclude these patients by design are not accurately representing the patient population for premenopausal hysterectomy. Application of this study design will allow for further innovation and exploration of the understudied research area of gynecologic health [13, 31].

### Supplementary Information

**Additional file 1.**

## Data Availability

The datasets generated and analyzed for the CHC study are not publicly available due to the terms of the data use agreement set by The North Carolina Translational and Clinical Sciences Institute (NC TRACS) who own and administer these data. The authors of this study cannot distribute these data to other entities due to the legal terms of this data use agreement. Access to the data for research purposes requires specific permissions from the NC TRACS. Researchers can place a data request at < https://tracs.unc.edu/index.php > Additionally, researchers can direct any data access questions to NC TRACS at <nctracs@unc.edu> or the corresponding author.

## Abbreviations

CHC: Carolina Hysterectomy Cohort
EMR: Electronic Medical Records
US: United States
AUB: Abnormal Uterine Bleeding
DUB: Dysfunctional Uterine Bleeding
SES: Socio-economic Status
IQR: Interquartile range
