# Supplementary material for: The Carolina hysterectomy cohort (CHC): a novel case series of reproductive-aged hysterectomy patients across 10 hospitals in the US south
Source: BMC Womens Health. 2023 Dec 19;23:674. doi: 10.1186/s12905-023-02837-8 (PMC10729499; doi:10.1186/s12905-023-02837-8)
Supplement: Supplementary file 1 — Additional file 1. [file 12905_2023_2837_MOESM1_ESM.pdf]

Additional Material 1: Administrative Billing Codes

This table provides the ICD-9/10 and CPT codes used to identify patient procedures and diagnoses.

**Code Type**	**Codes**
Hysterectomy	
ICD-9	68, 68.3, 68.31, 68.39, 68.4, 68.41, 68.49, 68.5, 68.51, 68.59, 68.6, 68.61, 68.69, 68.7, 68.71, 68.79, 68.9
ICD-10	OUT90ZL, OUT90ZZ, OUT94ZL, OUT94ZZ, OUT97ZZL, OUT97ZZ, OUT98ZL, OUT98ZZ, OUT9FZL, OUT9FZZ
CPT	56308, 58150, 58152, 58180, 58200, 58210, 58260, 58262, 58263, 58267, 58270, 58275, 58280, 58285, 58290, 58291, 58292, 58293, 58294, 58541, 58542, 58543, 58544. 58548, 58550, 58552, 58553, 58554, 58570, 58571, 58572, 58573, S2078
Pelvic Mass	
ICD-9	620, 620.1, 620.3-620.8, 620.9, 789.3, 789.3, 789.31, 789.32-789.37, 789.39
ICD-10	N83.0 - N83.9
Ectopic Pregnancy	
ICD-9	633, 633.01, 633.1, 633.11, 633.2, 633.21, 633.8, 633.81, 633.9, 633.91
ICD-10	O00.0-O00.91
Infertility	
ICD-9	628, 628.1-628.4, 628.8, 628.9, 629
ICD-10	N97, N97.0, N97.1, N97.2. N97.8, N97.9, E23.0, N88.3, None specified
Menopausal	
ICD-9	627, 627.1-627.4, 627.8, 627.9
ICD-10	N92.4, N95.0, N95.1, N95.2, E89.41, N95.8, N95.9
Prophylactic Surgery	
ICD-9	V50.49, V50.42, V50.41
ICD-10	Z40.00, Z40.01, Z40.02, Z40.09
Prolapse Incontinence	
ICD-9	618, 618.01-618.05, 618.09, 618.1-618.8, 618.81-618.84, 618.89, 618.9, 625.6, 788 788.3, 788.31, 788.33, 788.37, 788.38, 788.39, 788.4, 788.41, 788.63, 788.64, 788.65, 788.91
ICD-10	N81, N81.0, N81.10, N81.11, N81.12, N81.2-N81.6, N81.81-N81.85, N81.89, N81.9, N99.3, N39, N39.3, N39.4, N39.41, N39.45, N39.46, N39.49, N39.490, N39.491, N39.492, R35.0, R35.8, R39.15, R39.16, R39.11, R39.81
Miscellaneous	
ICD-9	614.7, 616, 616.1, 616.11, 616.8, 616.81, 616.89, 616.9, 619.1, 620, 620.1-620.9, 621, 621.1, 621.2, 621.4-621.9, 622, 622.2, 622.3, 622.4, 622.7, 622.8, 622.9, 623.8, 625.8, 625.9, 752, 752.1, 752.11, 789, 789.9
ICD-10	N72, N73.4, N73.9, N76, N76.0, N76.1, N76.2, N76.3, N76.81, N76.89, N77, N77.1, N76.5, N82.4, N83, N83.0, N83.00-N83.02, N83.1, N83.10-N83.12, N83.2, N83.20-N83.209, N83.291-N83.299, N83.3, N83.311-N83.319, N83.321-N83.329, N83.331-N83.339, N83.40-N83.42, N83.5, N83.511-N83.519, N83.521-N83.529, N83.53, N83.7, N83.8, N83.9, N84.0, N84.1, N85, N85.00, N85.2, N85.3-N85.9, N86, N88, N88.0, N88.1, N88.2, N88.8, N88.9, N89.8, N94.89, N94.9, Q50, Q50.4, Q50.5, Q50.6, R19, R19.8
Infectious	
ICD-9	567, 567.1, 567.2, 567.21, 567.22, 567.23, 567.29, 567.3, 567.31, 567.38, 567.39, 567.8, 567.81, 567.82, 567.89, 567.9, 568, 614, 614.1-614.5, 614.8, 614.9, 615, 639
ICD-10	K65.0, K65.1-K65.4, K65.8, K65.9, K66.0-K66.9, K67, K68, K68.11-K68.19, K68.9

Code Type	Codes
Hormonal Diagnosis	
ICD-9	256, 256.1, 256.2, 256.3, 256.31, 256.39, 256.4, 256.8, 256.9, 615.1
ICD-10	E28, E28.0, E28.1, E28.2, E28.3, E28.31, E28.319, E28.39, E28.8, E28.9, E89.40, E89.41, N71.1
Abnormal Bleeding	
ICD-9	626, 626.1-626.9
ICD-10	N91, N91.0-N91.5, N93, N93.0, N93.1, N93.8, N93.9
Endometriosis	
ICD-9	617, 617.1-617.6, 617.8, 617.9
ICD-10	N80, N80.0-N80.6, N80.8, N80.9
Pre-Cancerous Lesions	
ICD-9	233, 233.1, 233.2, 233.3, 233.31, 233.32, 233.39, 236, 236.3, 621.3, 621.31-621.35, 622.1, 622.11, 622.12, 795, 795.01-795.06, 795.08, 795.09, 795.1, 795.11, 795.12-795.15, 795.19
ICD-10	D06.0-D06.9, D07, D07.0, D07.1, D07.2, D07.3, D39, D39.0, D39.8, D39.9, N85.0, N85.00, N85.01, N85.02, N87, N87.0, N87.1, N87.9, R87.6, R87.61, R87.610-R87.615, R87.62, R87.620-R87.623, R87.628, R87.810, R87.811, R87.820
Benign Masses	
ICD-9	220, 221, 221.1, 221.2, 221.8, 221.9, 229
ICD-10	D27, D27.0, D27.1, D27.9, D28, D28.0, D28.1, D28.2, D28.7, D28.9, D36
Fibroids	
ICD-9	211.8, 215.6, 218, 218.1, 218.2, 218.9, 219, 219.1, 219.8, 219.9
ICD-10	D20.0, D20.1, D21.5, D25, D25.0 , D25.1 , D25.2, D25.9, D27, D27.0, D27.1, D27.9, D26, 26.0, D26.1, D26.7, D26.9, D28, D28.0, D28.1, D28.2, D28.7, D28.9
Pain	
ICD-9	338, 338.2, 338.28, 338.29, 338.4, 625, 625.1-625.5, 625.7, 625.71, 625.79, 789, 789.01-789.07, 789.09, 789.4, 789.41-789.47, 789.49, 789.6, 789.61-789.67, 789.69, 789.7
ICD-10	G89, G89.2, G89.29, G89.4, N94, N94.0, N94.10-N94.19, N94.2, N94.3, N94.4-N94.6, N94.8, N94.810-N94.819, N94.89, N94.9, R10, R10.10-R10.13, R10.2, R10.30, R10.33, R10.8, R10.811-R10.819, R10.821-R10.829, R10.83, R10.9
